# Moisturizer induced contact anaphylaxis

**DOI:** 10.1186/s13223-025-00954-7

**Published:** 2025-02-11

**Authors:** Bronte Jeffrey, Logan Gardner, Michelle Le, Julie Frost, Ming Wei Lin

**Affiliations:** 1 Department of Clinical Immunology, Westmead Hospital, Sydney, Australia; 2 St Vincent’s Clinical School, University of New South Wales, Sydney, Australia; 3 Faculty of Medicine and Health, University of Sydney, Sydney, Australia; 4 Department of Immunopathology, Westmead Hospital, Sydney, Australia; 5 Centre for Immunology and Allergy Research, Westmead Institute of Medical Research, Sydney, Australia; 6 Allergy, Asthma and Clinical Immunology, The Alfred Hospital, Melbourne, Australia; 7 School of Public Health & Preventive Medicine, Monash University, Melbourne, Australia

**Keywords:** Allergy, Contact, Anaphylaxis, Hypersensitivity, Urticaria

## Abstract

**Background:**

Contact allergens typically trigger localised reactions, but systemic Type I hypersensitivity from skin contact reactions are rare.

**Case presentation:**

We present the case of a 69-year-old non-atopic male who developed anaphylaxis following the application of moisturizer to an area of chemical burns. Skin testing showed a strong positive result to moisturizer. Whilst not all ingredients were available for testing, phenoxyethanol was thought to be the likely culprit agent based on literature review and a weakly positive skin test result.

**Conclusion:**

Products such as moisturizers can rarely trigger anaphylaxis, especially when applied to damaged skin which may favor systemic absorption. This case highlights the need for careful consideration of cosmetic application when discerning culprit allergens.

## Background

Whilst contact anaphylaxis is rarely encountered in clinical practice, its true incidence is uncertain as existing literature is limited to case reports. Contact anaphylaxis represents the severe end of the spectrum of immunological contact urticaria and is thought to be a Type I hypersensitivity reaction, whereby antigens absorbed through the dermal barrier bind with specific IgE molecules on pre-sensitized mast cells, resulting in mast cell degranulation with the release of histamine and other vasoactive substances, such as prostaglandins, leukotrienes and kinins [1]. This can result in clinical manifestations ranging from localised, milder symptoms (pruritis, paresthesia and localised wheal and flares) to more generalised urticaria or extracutaneous manifestations, including angioedema, bronchospasm, diarrhoea or severe anaphylaxis [2]. This has been best described in relation to grains and natural rubber latex [3]. Contrastingly, non-immunological contact urticaria is more common and is the result of direct mast cell degranulation and release of localised vasogenic mediators, but rarely results in systemic symptoms [4]. This latter type of urticaria is typically not responsive to antihistamines and has been described in relation to numerous substances, including benzoic acid, sorbic acid and dimethyl sulfoxide [3, 4].

## Case presentation

We present the case of a 69-year-old non-atopic male who developed anaphylaxis following the application of Redwin Moisturizer. The patient had suffered extensive chemical burns from home concrete agents which required daily dressing. The patient applied a range of different moisturizers to the affected area which did not elicit any symptoms of concern. Within minutes of the first application of the Redwin moisturizer to his leg, the patient experienced immediate paresthesia at the site, flushing and had a syncopal episode. He was brought to hospital via ambulance and emergent treatment included fexofenadine and intravenous fluids, with symptoms resolving over 4 h. No adrenaline was administered; however, tryptase taken at the time of the event was elevated at 31.9 micrograms/L (normal reference range 0-11.4 micrograms/L). Tryptase in convalescence (3 months later) was 5.9 micrograms/L, suggesting this was an anaphylactic reaction. No other exposures within hours preceding the reaction, including food, drugs, topical products or insect stings, were reported. Similarly, other cofactors, such as recent viral illness or NSAID use, were not identified.

The patient’s background history was significant for a single episode of an unspecified pruritic, skin rash 15 years prior, self-attributed to a new laundry detergent given the distribution of the rash which developed in areas of contact with clothing. This lasted several weeks and subsequently resolved without recurrence. There is no history of atopy or allergy otherwise.

**Fig. 1:**
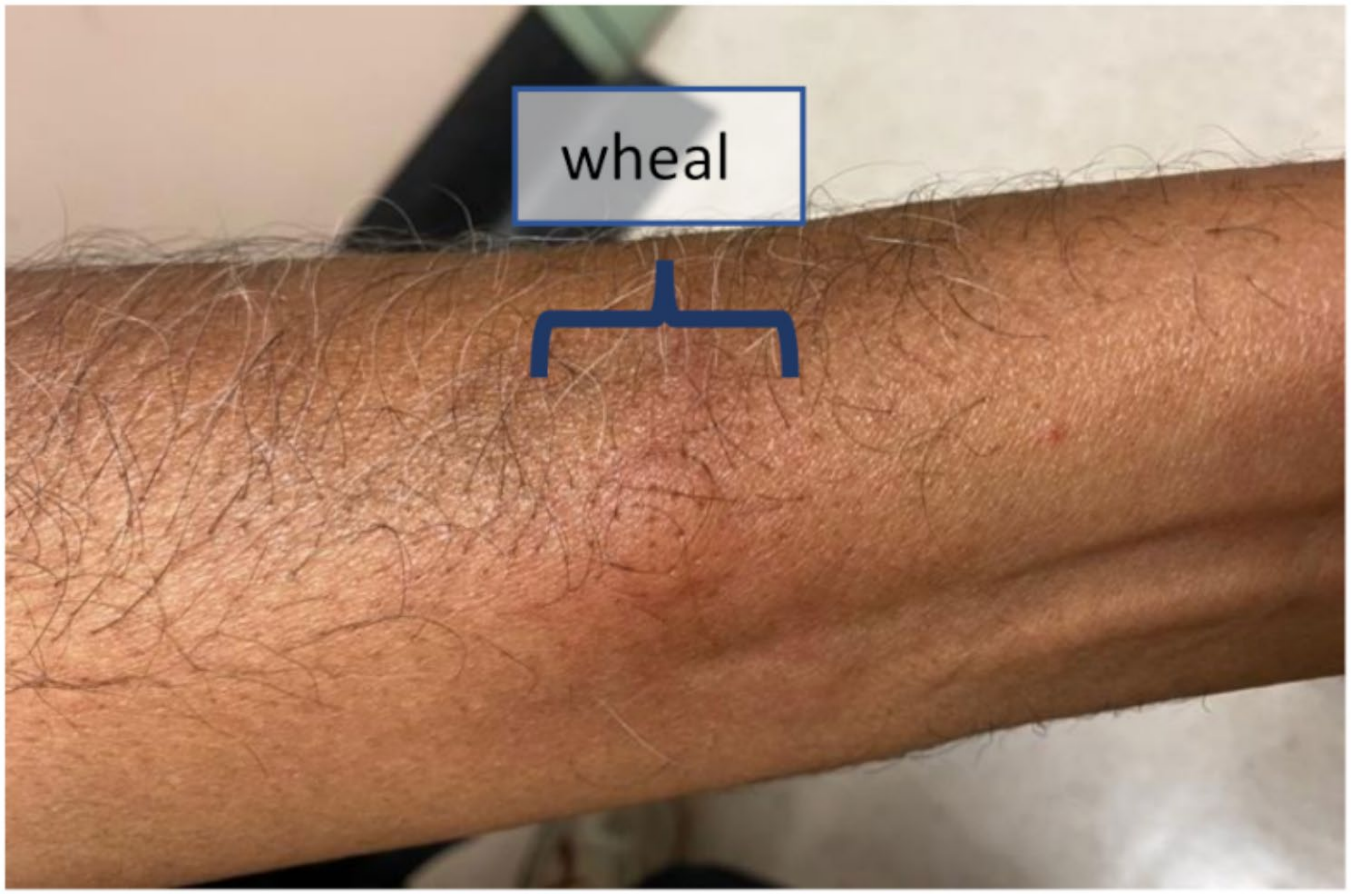
Skin prick test demonstrating wheal from implicated moisturizer

Skin prick testing (SPT) was performed using a dilution 1:2 of Redwin moisturizer in normal saline and the patient’s wife was used as a “negative control” to exclude an irritant effect of the product. The patient developed a wheal of 13 × 10 mm after 15 min, with the control developing no reaction (Fig. 1). Further testing was performed with neat phenoxyethanol as it was identified as a potential culprit when comparing the tolerated QV moisturizer (tolerated both before and after the reaction) with Redwin moisturizer (Table 1). The patient developed a 3 × 4 mm wheal, with again the control subject developing no wheal. Of the other ingredients present in Redwin but not QV moisturizer (Table 1, blue), only p-chloro-m-Cresol has been reported as an allergen causing a Type I hypersensitivity reaction which was contact urticaria but not anaphylaxis [2]. This product was not available for skin prick testing at our institution. Furthermore, Triticum Vulgare (wheat) Germ Oil was judged unlikely as possible causative allergen as the patient was able to tolerate a wheat containing diet. Five other ingredients, that could not be skin tested, were found in the Redwin moisturizer and not in the tolerated QV moisturizer (Table 2). Although some may rarely cause allergic contact dermatitis, there are no descriptions of immediate-type hypersensitivity to these agents. Additionally, there was no evidence of a *c-KIT* mutation in peripheral blood to suggest an underlying mast cell disorder.

**Table 1:**
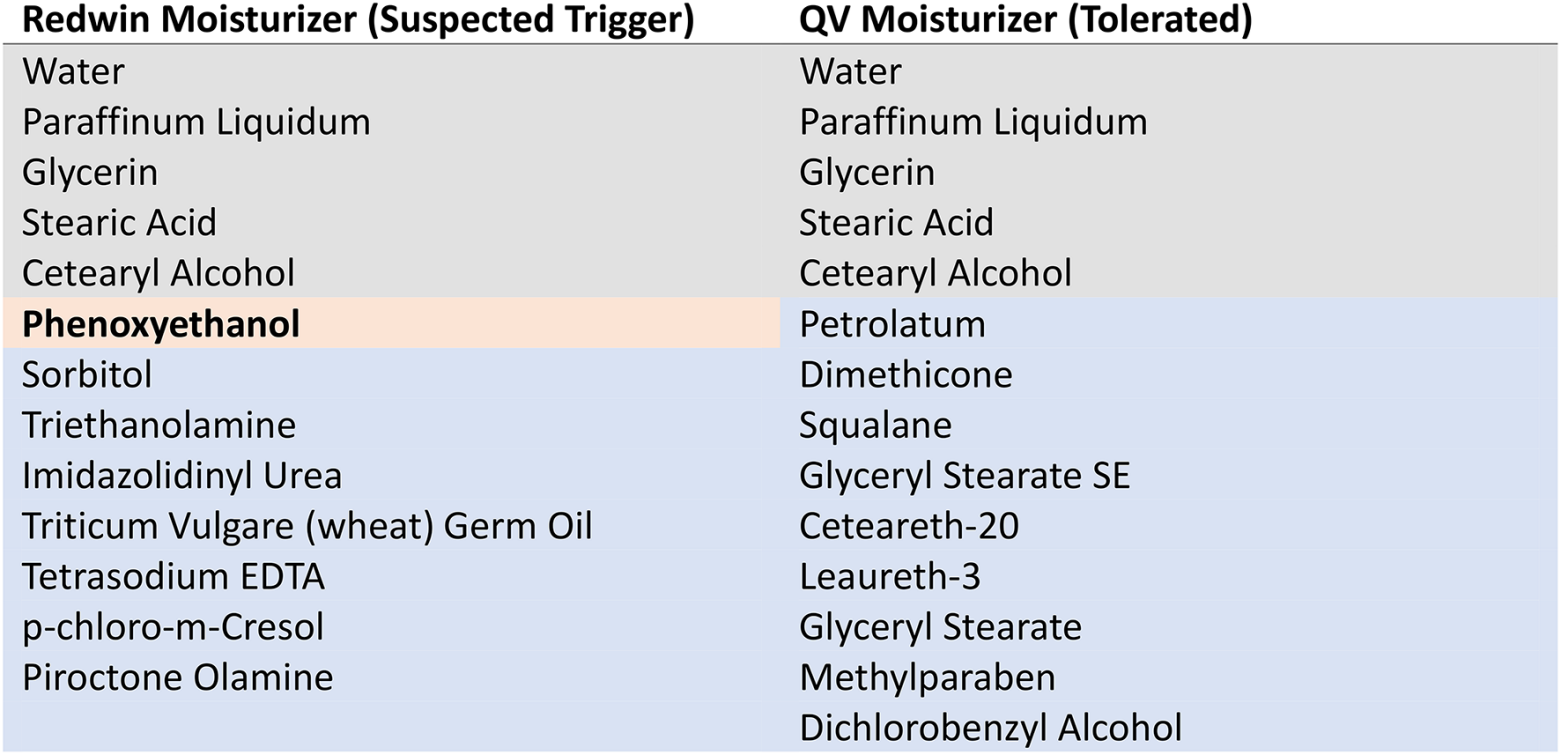
Ingredients in Redwin Moisturizer and QV moisturizer. Grey – shared ingredients. Orange – implicated ingredient. Blue – different ingredients

**Table 2:** Sensitization studies in Redwin ingredients not able to be tested

Ingredient	Evidence
Sorbitol	No specific sensitization study performed. Structurally similar simple sugar alcohols Mannitol and Xylitol did not demonstrate skin reactions or sensitization in patch testing studies [6]. Sorbitol derivatives (e.g., sorbitan sesquioleate) have patch testing data to suggest these can be sensitizing [7].
Triethanolamine	Analysis of 85,098 patients who were patch tested with Triethanolamine 2.5% petrolatum reported a positivity rate 0.4% of patients. The profile of reactions, however, suggested a slight irritant potential rather than true allergic response in most cases [8].
Imidazolidinyl Urea	A retrospective study of 6845 patients patch tested with imidazolidinyl urea demonstrated a positivity rate of 1.9% [9]. There are case reports of contact dermatitis from products containing Imidazolidinyl Urea [10, 11]
Triticum Vulgare (wheat) Germ Oil	Patch test of powder containing 13% Triticum Vulgare (Wheat) Germ Extract was reported in 105 human subjects. No irritation or sensitization was observed [12].
Tetrasodium EDTA	Skin sensitization studies (including a repeat-insult patch test) of products including EDTA did not demonstrate sensitization [13]. Despite this, there is a case report of contact allergy to tetrasodium EDTA [14].
p-chloro-m-Cresol	Patch testing of 3062 patients across 7 centres in the UK demonstrated a sensitization rate of 0.6% [15]. Two case reports of contact urticaria, but no systemic reactions reported [16, 17].
Piroctone Olamine	Animal sensitization studies conducted on guinea pigs did not demonstrate sensitization [18]

The patient was subsequently advised to avoid products with phenoxyethanol as the most likely culprit ingredient based on literature review and available testing. This included avoiding vaccines such as Vivaxim (Hepatitis A-typhoid), Quadracel (diptheria, tetanus, acellular pertussis, inactivated poliovirus) or other vaccines containing phenoxyethanol until further immunology review could be conducted. If required, vaccine specific skin testing could be performed in future as previously described in the literature [5]. The limitations of not being able to test all individual ingredients in the moisturizer was communicated to the patient and an adrenaline auto-injector was prescribed in the possible event of future reactions. The reaction was also reported to the relevant governing authorities.

## Discussion

Phenoxyethanol has a wide range of antimicrobial activity against gram positive bacteria, gram negative bacteria and yeasts and is, thereby, commonly used as a preservative in cosmetic, cleaning, laundry and craft products [19]. Prevalence across cosmetic products is estimated between 14 and 43% [19]. Most governing bodies limit a concentration of 1% in cosmetics. The concentration in Redwin moisturizer is not reported.

Phenoxyethanol has rapid percutaneous absorption, regardless of the concentration. This does not bind or accumulate in the skin and very little (< 0.1%) remains after 24 h of exposure. After absorption, it is metabolised by either the skin or liver into the major metabolite 2-phenoxyacetic acid and, thereafter, excreted in the urine [19].

Animal models have not demonstrated evidence of sensitization to phenoxyethanol. In human studies, phenoxyethanol is considered a “rare” allergen. A retrospective study including 6,932 patch tests assessing presence of Type IV hypersensitivity reactions to phenoxyethanol at a concentration of 1% had a positivity rate of only 0.24% [19]. This is consistent with other reviews of patch testing and phenoxyethanol is not currently classified as a sensitizer by the European Chemicals Agency (ECHA).

In the literature, there is an increasing number of case reports of contact urticaria and other localised reactions, such as angioedema, associated with phenoxyethanol [20–25]. A review of cosmetic components causing contact urticaria attributes this reaction to an immunological (IgE) mediated mechanism [2]. IgE specific for phenoxyethanol has never been isolated, however, and other authors favour a non-immunological pathogenesis [19]. There is only a single case of contact anaphylaxis attributed to phenoxyethanol where the patient had a 6-month history of urticaria to cosmetic products, culminating in a reaction including urticaria, rhinorrhea, dyspnoea and presyncope [5].

Whilst we judge that phenoxyethanol is the most likely allergen in the Redwin moisturizer based on literature review and a weakly positive skin, we cannot rule out that another component of the moisturizer, that was not tested, is responsible. Furthermore, in our patient, two potential co-factors are plausible which may have increased the severity of the reaction. Firstly, p-chloro-m-Cresol and tetrasodium EDTA have been reported to increase the penetration of other cosmetic ingredients which may have increased absorption of the phenoxyethanol [26]. Presence of a chemical co-factor, such as p-chloro-m-Cresol, could provide an explanation for the increased size of the moisturizer SPT wheal compared to the phenoxyethanol SPT result. Secondly, our patient had an impaired skin barrier due to the presence of chemical burns which similarly may have increased absorption of the allergen, but also allowed the immunological defence mechanisms provided by the skin to be bypassed. We have not identified phenoxyethanol in any other products or medicines he uses, although we hypothesise given the common inclusion of phenoxyethanol in many products that there may have been a previous sensitizing event not recalled by the patient, including the duration when the chemical burn was present.

## Conclusion

Contact hypersensitivity is likely an underappreciated mechanism of anaphylaxis in clinical practice. This case highlights the need for careful consideration of cosmetic application when discerning culprit allergens, even in presentations with anaphylaxis. In this case, an impaired skin barrier may have been a sensitizing event and augmented the severity of the reaction.

## Data Availability

No datasets were generated or analysed during the current study.
